# Bacteria-Produced Algicide for Field Control of Toxic Dinoflagellates Does Not Cause a Cortisol Stress Response in Two Estuarine Fish Species

**DOI:** 10.1007/s10126-024-10383-z

**Published:** 2025-01-14

**Authors:** Victoria E. Simons, Timothy E. Targett, Patrick M. Gaffney, Kathryn J. Coyne

**Affiliations:** College of Earth, Ocean and Environment, School of Marine Science and Policy, University of Delaware, Lewes, DE 19958 USA

**Keywords:** Algicide, Algal blooms, Fish, Cortisol, Stress response, Multiple stressors

## Abstract

Application of algicides produced by naturally occurring bacteria is considered an environmentally friendly approach to control harmful algal blooms. However, few studies assess the effects of bacterial algicides on non-target species, either independently or with other stressors. Here, we measured sub-lethal effects of dinoflagellate-specific algicide IRI-160AA on the estuarine fish *Fundulus heteroclitus* and *Menidia menidia* in laboratory experiments. Plasma cortisol levels were measured to test whether a neuroendocrine stress response was induced in these fish following exposure to the algicide alone, and in combination with diel-cycling hypoxia and/or pH, at 25 and 30 °C. Results show that exposure to IRI-160AA does not significantly affect cortisol levels in either species, at either temperature tested, whether exposure occurs independently or with co-occurring hypoxia and/or pH cycles as potential multiple stressors. These results support the application of IRI-160AA as an environmentally friendly approach to control harmful algal blooms in estuarine environments.

**Supplementary Information:**

The online version contains supplementary material available at 10.1007/s10126-024-10383-z.

## Introduction

Harmful algal blooms (HABs) have been increasing in frequency and intensity in coastal regions worldwide, with both ecological and socio-economic impacts (Anderson et al. 2021; Wells et al. 2015). Among the most toxigenic HABs are those caused by dinoflagellates that produce potent toxins, with the potential to kill fish and other marine organisms and/or become concentrated in higher trophic levels (Anderson et al. 2021). Research and management efforts have focused on development of environmentally friendly approaches to manage these blooms, including the application of naturally occurring algicidal bacteria (reviewed by Coyne et al. 2022). Hare et al. (2005) described the algicidal activity of one such bacterial strain designated *Shewanella* sp. IRI-160, isolated from coastal waters of the mid-Atlantic region of the USA. Subsequent research showed that *Shewanella* sp. IRI-160 does not require direct contact to induce cell death in dinoflagellates and that algicidal compounds were retained in the bacteria-free algicidal filtrate, termed IRI-160AA (Pokrzywinski et al. 2012). The filtrate from *Shewanella* sp. IRI-160 includes a suite of amines shown to act synergistically to inhibit dinoflagellate growth (Ternon et al. 2019; Johnson 2023; Wang and Coyne 2024). IRI-160AA was effective against all dinoflagellates tested, with no negative effects on other phytoplankton species (Pokrzywinski et al. 2012; Tilney et al. 2014) or microzooplankton grazing rates (Grasso 2019). Further laboratory experiments demonstrated no adverse effects of IRI-160AA on invertebrates including copepod *Acartia tonsa* (nauplii and adults), blue crab *Callinectes sapidus* (megalopa postlarvae), and eastern oyster *Crassostrea virginica* (pediveliger larvae), and only transient effects were noted for activity in zoea life stages of *C. sapidus* after IRI-160AA exposure (Simons et al. 2021).

In this study, we examined sub-lethal effects of IRI-160AA in two fish species. Mummichog (*Fundulus heteroclitus*) and Atlantic silverside (*Menidia menidia*) are abundant in shallow estuarine and inshore marine environments along the USA east coast where they are important to ecosystem energy transfer and serve as common food sources for many birds and larger fish (Abraham 1985; Fay et al. 1983; Nemerson and Able 2004; Torre and Targett 2017). These species co-occur in salt marsh estuaries that experience harmful dinoflagellate blooms (e.g. Tilney et al. 2014). They are often exposed to environmental stresses due to high summer temperature and also diel (24 h) cycles of nighttime hypoxia with high pCO_2_ (low pH) and daytime hyperoxia with low pCO_2_ (high pH) caused by net respiration-photosynthesis cycles (Baumann et al. 2015; Baumann and Smith 2018; Tyler et al. 2009). There is a growing understanding that multiple stressors experienced simultaneously need to be considered when examining ecological impacts of natural and anthropogenic conditions (Baumann 2019; Breitburg et al. 2019; Gobler and Baumann 2016; Gunderson et al. 2015; McBryan et al. 2013).

It is essential to consider the potential impacts of IRI-160AA application in areas inhabited by these fish, within this multiple stressor context. Primary stress responses in fish typically occur within minutes after exposure to stressor(s), resulting in the release of hormones, particularly cortisol, into the bloodstream (Alfonzo et al. 2020; Barton 2002; Lawrence et al. 2018; Pankhurst 2011). Indeed, elevated cortisol is a well-established indicator of the degree of stress experienced by fish (Alfonzo et al. 2020; Barton 2002; Barton and Iwama 1991; Schulte 2014). Cortisol initiates a cascade of downstream physiological and behavioral responses, causing a diversion of resources with impacts on whole-animal performance that can lead to reductions in fitness and ultimately survival (Barton 2002; Busch and Hayward 2009; Birnie-Gauvin et al. 2017; Bordin and Freire 2021).

The purpose of this investigation was to: (1) experimentally determine whether exposure to IRI-160AA, when added at a level that would control dinoflagellate growth, caused a significant cortisol stress response in these fish and (2) whether diel DO and pH cycles, either co-occurring or individually, functioned as multiple stressors to exacerbate the cortisol stress response. Laboratory experiments were conducted on *F. heteroclitus* and *M. menidia* exposed to IRI-160AA, with and without added potential stressors (diel-cycling hypoxia and/or pH), at two temperatures. Plasma cortisol levels were measured to quantify neuroendocrine stress response.

## Materials and Methods

Fish were caught in Lewes, DE, and held in laboratory holding trays (2.5 m × 0.13 m) of air-saturated recirculating 25‰ seawater. They acclimated for at least 7 days to 25 or 30 °C and 14:10-h light:dark photoperiod. Fish were fed frozen mysid shrimp (*Mysis relicta*) twice daily. Large individuals (*F. heteroclitus*: ~ 7–14 g, ~ 80–100-mm total length (TL), mean 86 (± 0.5 SE) mm; *M. menidia*: ~ 3–15 g, ~ 60–110 mm TL, mean 91 (± 1.4 SE) mm) were selected for experiments to maximize blood volume for cortisol assays.

Algicide IRI-160AA was prepared in f/2 + CAA media as described in Simons et al. (2021). Ammonium concentration in IRI-160AA was lowered by addition of Ammonium Neutralizing Crystals (Spectrum Brands Pet, LLC, Blacksburg, VA) and measured using the salicylate method (Kempers and Kok 1989). IRI-160AA was then filtered through a 0.2-µm polycarbonate filter to remove any residual solids. The half-maximal effective concentration (EC50) of the batch of algicide used here was estimated to be 1% (v/v) in bioassays using the dinoflagellate *Karlodinium veneficum* as described in Pokrzywinski et al. (2012).

Fish exposure experiments were conducted in five recirculating aquarium systems (~ 400 L), each with ten ~ 20-L tanks containing 25‰ seawater (Online Resource 1). Seawater in each system circulated through the 10 tanks and surrounding tray, into a sump, and back to the tanks and tray. A computer controlled the DO and pH (NBS scale) in each aquarium system using a program written in LabVIEW instrumentation software (2010 version 10.0.1 SP1) interfaced with a Hach sc200 Universal Controller, Hach LDO dissolved oxygen probe, and Hach Differential pH/ORP sensor. Every ~ 5 min, a small amount of water from each system was diverted over the DO and pH sensors. If DO or pH differed from that specified by the program for time-specific set points, O_2_, N_2_, CO_2_, or CO_2_-free air was added to that system to adjust levels and maintain desired treatment conditions.

Dissolved oxygen and pH data from all aquarium systems were saved by the program to create continuous records throughout experiments. Readings from DO and pH sensors were confirmed twice-daily with calibrated handheld instruments (YSI ProDSS with DO sensor and Fisher Scientific AB15 Plus meter with accuTupH combination pH and temperature electrode). Adjustments to the continuous record were made as necessary.

Four experimental runs were conducted on each species (*Fundulus*: Runs F1-F4, *Menidia*: Runs M1-M4), two at 25 °C and two at 30 °C, and each run had five treatments (Tables 1 and 2). The algicide treatment consisted of IRI-160AA (cell-free filtrate after incubating *Shewanella* sp. IRI-160 in f/2 + CAA media), which was added to fish tanks at 1% (v/v) (EC50 algicide concentration from the *K. veneficum* bioassays). The media control treatment was f/2 + CAA media, without algicide, added at the same concentration as in the algicide treatment. The first set of experiments for each species at each temperature (Table 1; Runs F1, F3, M1, and M3) consisted of (1) seawater control (C) with static DO and pH (Table 2) and no algicide or media, (2) diel-cycling pH treatment (d-cpH), (3) diel-cycling pH plus algicide treatment (d-cpH + A), (4) diel-cycling DO treatment (d-cDO), and (5) diel-cycling DO plus algicide treatment (d-cDO + A). The second set of experiments for each species at each temperature (Table 1; Runs F2, F4, M2, and M4) consisted of (1) another seawater control (C) as above, (2) media control (M) with static DO and pH, (3) algicide treatment (A) with static DO and pH, (4) diel-cycling DO and pH treatment (d-cDOpH), and (5) diel-cycling DO and pH plus algicide treatment (d-cDOpH + A).

**Table 1:** Treatment conditions for *Fundulus heteroclitus* (runs F1–F4) and *Menidia menidia* (runs M1–M4). Runs 1 and 2, for each species, were conducted at 25 °C and runs 3 and 4 at 30 °C. Salinity in all experiments was 25‰. Treatments with algicide refer to the algicide IRI-160AA prepared in media (f/2 + CAA) and added at 1% v/v, the half-maximal effective concentration (EC50) value for the target dinoflagellate *Karlodinium veneficum* (see text). Treatment conditions are defined by Xs in the columns beneath the abbreviations used for the five treatments tested (Table 2) in each panel of Figs. 1 and 2. Runs included as follows: *C*, seawater control with static DO and pH (Table 2); *M*, algicide-free media control; *A*, algicide treatment; *d-cpH*, diel-cycling pH treatment; *d-cpH* + *A*, diel-cycling pH plus algicide treatment; *d-cDO*, diel-cycling DO treatment; *d-cDO* + *A*, diel-cycling DO plus algicide treatment; *d-cDOpH*, diel-cycling DO and diel-cycling pH treatment; *d-cDOpH* + *A*, diel-cycling DO and diel-cycling pH plus algicide treament

Treatment conditions	F1 and F3 & M1 and M3					F2 and F4 & M2 and M4				
	C	d-cpH	d-cpH + A	d-cDO	d-cDO + A	C	M	A	d-cDOpH	d-cDOpH + A
Algicide			X		X			X		X
DO—static control	X	X	X			X	X	X		
DO—diel-cycling				X	X				X	X
pH—static control	X			X	X	X	X	X		
pH—diel-cycling		X	X						X	X
Media—control							X

**Table 2:**
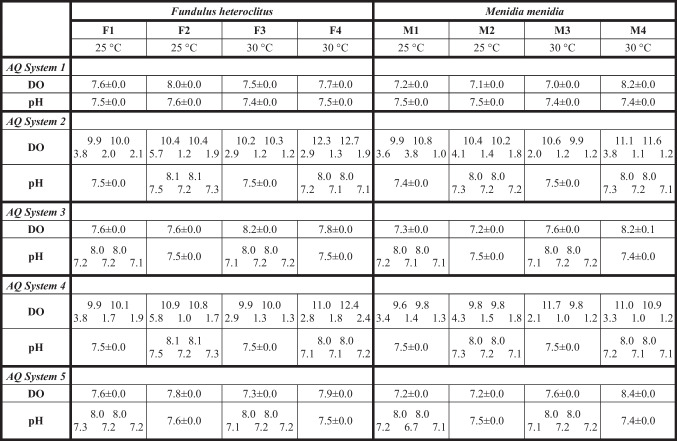
Dissolved oxygen (DO; mg O_2_ L^-1^) and pH (NBS scale) values during the four experimental runs conducted on *Fundulus heteroclitus* (runs F1-F4) and *Menidia menidia* (M1-M4) at 25 and 30 °C. Mean (± SE) DO and pH are shown across tanks for static treatments and as minimum and maximum values for diel-cycling treatments. Cells reporting diel-cycling DO and pH treatments show two high numbers above three low numbers. These are, from left to right, the low-to-high-to-low-to-high-to-low extremes experienced over the ~ 48 h of diel-cycling treatment conditions. Means were calculated from the continuous DO and pH records using values from ~ 09:00 h on the second day, when computer control of diel-cycling conditions started, until ~ 09:00 h on the fourth day when fish were anesthetized. Aquarium (**AQ**) **System 1** was the static DO and pH control for each run, **AQ System 2** was the diel-cycling DO with static pH for runs 1 and 3, and the diel-cycling DO with associated diel-cycling pH for runs 2 and 4. **AQ System 3** was static DO with diel-cycling pH for runs 1 and 3, and the algicide treatment with static DO and pH for runs 2 and 4. **AQ System 4** was diel-cycling DO with static pH plus algicide for runs 1 and 3, and the diel-cycling DO with associated diel-cycling pH plus algicide for runs 2 and 4. **AQ System 5** was static DO with diel-cycling pH plus algicide for runs 1 and 3, and the media control with static DO and pH for runs 2 and 4

Diel-cycling DO and pH treatment conditions were chosen to represent the magnitude and rate of diel DO and pH cycles known to occur in shallow salt marsh tributaries in the US Mid-Atlantic and South Atlantic Bights (Baumann and Smith 2018; Cochran and Burnett 1996; Tyler et al. 2009; Ullman et al. 2013). DO cycles in experiments averaged 2.0–10.6 mg O_2_ L^−1^ and pH cycles averaged 7.2–8.0 (Table 2). Both cycled between minimum and maximum values at the beginning of the light (07:00 h) and dark (21:00 h) periods, respectively.

On the first day of each experiment, fish were moved from holding trays to the five experimental aquarium systems (Online Resource 1) at 08:00 h. Each of the 10 tanks per aquarium system held one fish at their acclimation temperature, salinity, and photoperiod. The following day, for the diel-cycling treatments, computer control of diel-cycling DO and/or pH conditions began at ~ 09:00 h and continued through day 4. On the fourth day, algicide treatment, media control, or seawater control was added to the appropriate system (Table 1). This process started at ~ 08:00 h (1 h after lights on and 1 h after minimum DO and/or pH in the diel-cycling treatments) for the first aquarium system, and at 15-min intervals for subsequent systems. At ~ 09:00 h, fish were removed from tanks in the first aquarium system and immediately anesthetized with MS-222 (~ 0.1 g L^−1^ seawater). Blood (~ 20–100 µL) was drawn from the primary caudal vasculature with heparinized capillary tubes and quickly placed in labeled microcentrifuge tubes on ice. Fish length (mm TL) and time from placement into anesthetic until blood was on ice (handling time) were recorded for each fish. Fish were immediately killed by severing the cervical spinal cord, followed by pithing. The process was repeated for the second through fifth aquarium systems. This protocol was approved by IACUC (AUP: 1302–2017-A) at the University of Delaware.

Blood was centrifuged at 5000 g at 4 °C for 10 min. Plasma was removed and stored at − 80 °C until analysis. Cortisol (ng mL^−1^) was measured using Cortisol ELISA kits (ADI-901–071; Enzo Life Sciences, Inc., Farmingdale, NY, USA) with established procedures (Cousineau et al. 2014; Sadoul and Geffroy 2019).

### Data Analyses

Effects of treatments on cortisol levels were analyzed using linear models, with treatments as categorical variables and fish size (mm TL) and handling time (minutes) as covariates. Type II sums of squares were calculated using the car package in R (R Statistical Software v4.4.0; R Core Team 2024). Response variables were transformed if needed to meet assumptions of the linear model following Box-Cox and residual analyses. Experimental runs were conducted independently in the laboratory and were analyzed separately to examine cortisol levels in the algicide treatment alone, algicide in combination with diel-cycling DO and/or pH treatments, and concurrently run controls. One outlier was removed from the d-cDO treatment in Run M3 for *M. menidia*. This cortisol value was more than twice the next highest value and well above cortisol values generally reported for other fish (Barton 2002).

For each of the eight experimental runs (F1–F4 and M1–M4), an omnibus ANCOVA was conducted to test for differences in cortisol levels across the five conditions examined (Tables 1 and 2). When significant variation was observed, homogeneous subsets were identified by removing the group with the most deviant mean and repeating the ANCOVA. For all runs, homogeneous subsets could be determined by removing no more than one treatment group.

## Results

**Fig. 1:**
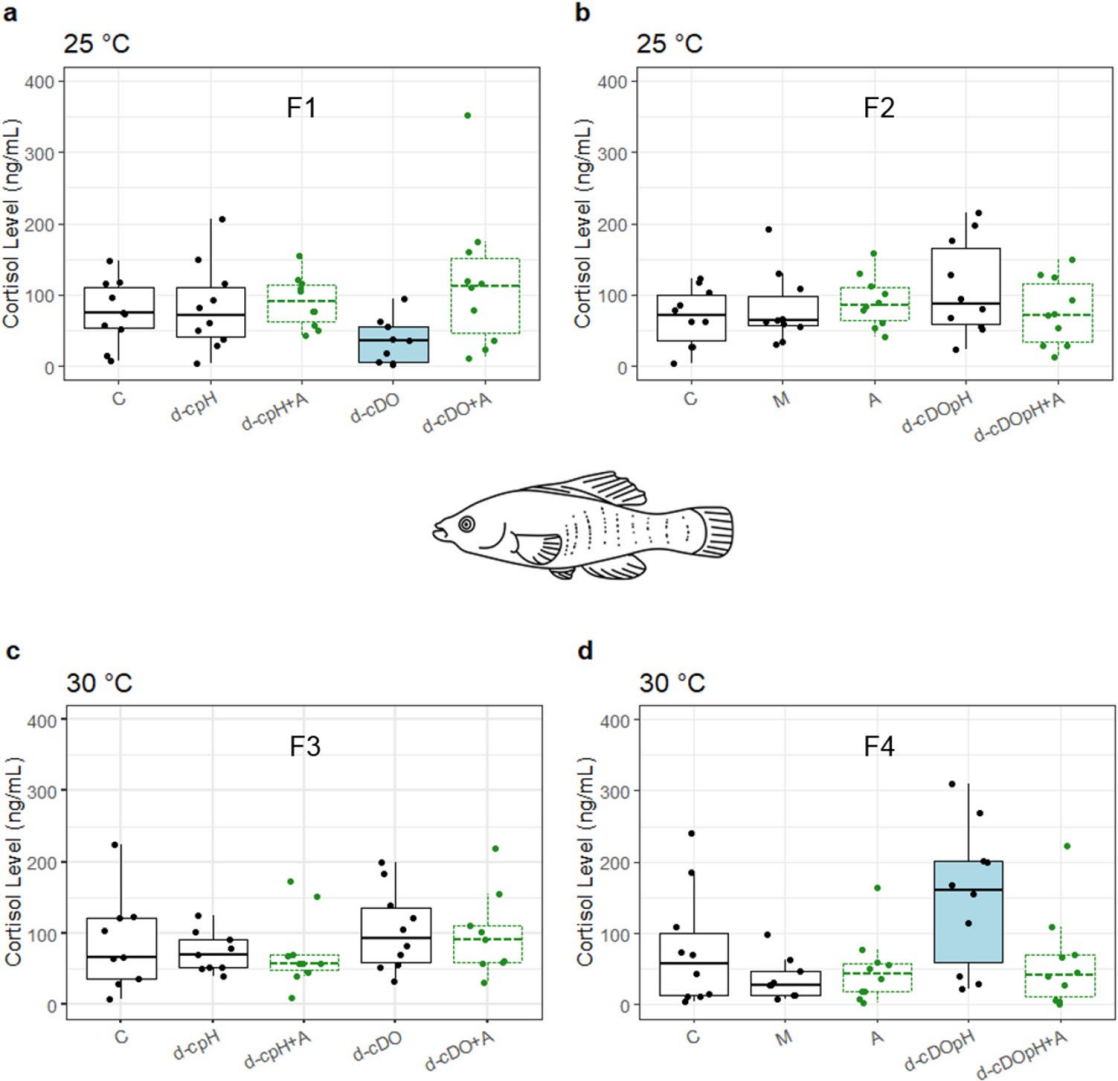
Box plots of plasma cortisol levels (ng mL^−1^) from *Fundulus heteroclitus* exposed to control and treatment conditions at 25 °C (panels **a** and **b**) and 30 °C (panels **c** and **d**). Tukey-style box plots show medians as horizontal lines within boxes, which enclose the middle 50% of the data (25th to 75th percentile), and data points are individual dots. Vertical lines (“whiskers”) represent the extent of data points within 1.5 times the interquartile range (distance from the 25th to 75th percentile), whereas points beyond the whiskers represent more extreme values. Labels F1–4 refer to the four experimental runs for *F. heteroclitus* in Tables 1 and 2. Treatments containing algicide have medians with dashed lines. In each panel, treatments within the primary homogeneous subset have unshaded boxes; and significantly different treatments, if any, are shaded. See Table 1 for treatment abbreviations. Median number of fish assayed per treatment was 10 (range = 9–10)

There was no evidence that algicide IRI-160AA treatment (A) affected cortisol level of *F. heteroclitus* at either temperature, as cortisol levels were not significantly different from the seawater control (C) nor the media control (M) (Fig. 1b and d). All three of these treatments had static DO and static pH at control levels (Tables 1 and 2).

Cortisol level of *F. heteroclitus* exposed to algicide plus diel-cycling pH (d-cpH + A) was not significantly different from the corresponding diel-cycling pH treatment without algicide (d-cpH) at either temperature (Fig. 1a and c). And although cortisol in the algicide treatment with diel-cycling DO (d-cDO + A) was significantly higher than in the corresponding treatment without algicide (d-cDO) at 25 °C, this was because cortisol in the d-cDO treatment was significantly lower than the primary homogeneous subset (Fig. 1a). Furthermore, the d-cDO + A treatment was not significantly different than the seawater control at either temperature (Fig. 1a and c).

Cortisol level of *F. heteroclitus* exposed to algicide IRI-160AA in combination with both diel-cycling hypoxia and pH (d-cDOpH + A) was not elevated compared to the d-cDOpH treatment without algicide, at either temperature (Fig. 1b and d). In fact, the cortisol level in d-cDOpH + A was significantly lower than the corresponding treatment without algicide at 30 °C (Fig. 1d). Further evidence that algicide exposure does not interact with these potential multiple stressors to elevate cortisol in *F. heteroclitus* is provided by the lack of significant difference in cortisol level between fish exposed to the algicide treatment and the d-cDOpH + A treatment, at either temperature (Fig. 1b and d).

**Fig. 2:**
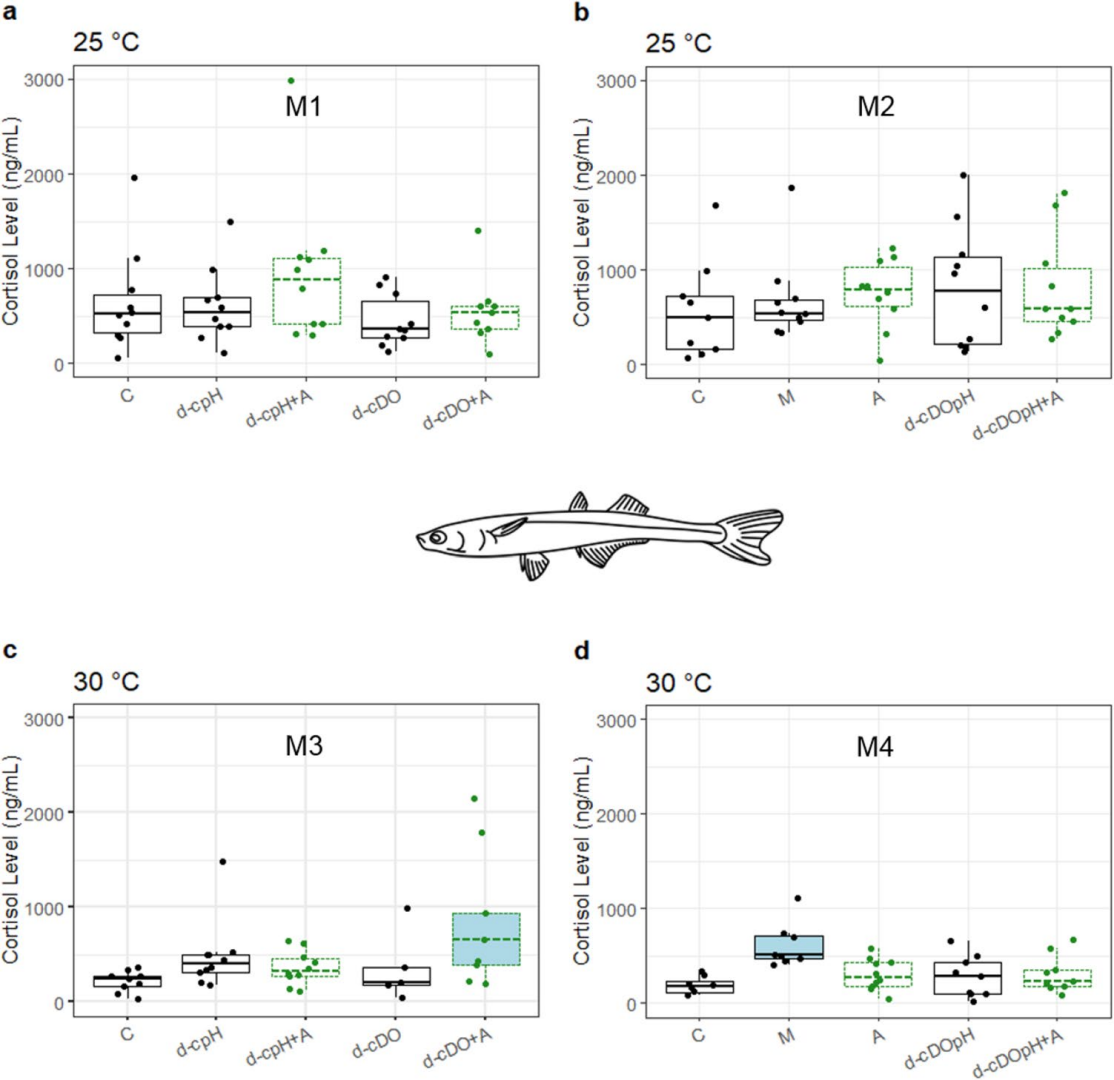
Box plots of plasma cortisol levels (ng mL^−1^) from *Menidia menidia* exposed to control and treatment conditions at 25 °C (panels **a** and **b**) and 30 °C (panels **c** and **d**). Tukey-style box plots show medians as horizontal lines within boxes, which enclose the middle 50% of the data (25th to 75th percentile), and data points are individual dots. Vertical lines (“whiskers”) represent the extent of data points within 1.5 times the interquartile range (distance from the 25th to 75th percentile), whereas points beyond the whiskers represent more extreme values. Labels M1–4 refer to the four experimental runs for *M. menidia* in Tables 1 and 2. Treatments containing algicide have medians with dashed lines. In each panel, treatments within the primary homogeneous subset have unshaded boxes; and significantly different treatments, if any, are shaded. See Table 1 for treatment abbreviations. Median number of fish assayed per treatment was 10 (range = 5–10). Only one treatment (d-cDO in panel **c**) had fewer than eight fish. One outlier value was removed from the d-cDO treatment in panel **c**; see “Materials and Methods” section

Similar to *F. heteroclitus*, cortisol level of *M. menidia* exposed to IRI-160AA (A) was not significantly different from the seawater control (C) at either temperature, nor from the media control (M) at 25 °C (Fig. 2b and d). Cortisol level in M at 30 °C, however, was significantly elevated above C (Fig. 2d). As this elevated cortisol was not associated with an elevation in A (also containing media), there is no evidence that media had an effect on observed algicide results.

Treatments that measured cortisol level of *M. menidia* exposed to algicide in addition to diel-cycling hypoxia and/or pH showed no significant cortisol elevation at 25 °C (Fig. 2a and b). The only significant difference in cortisol levels across these treatments at 30 °C was the significantly higher cortisol seen in the d-cDO + A treatment (Fig. 2c). This is the only case among the six comparisons of algicide-containing DO and/or pH treatments with corresponding treatments without algicide for this species in which a difference in cortisol level was evident. Importantly, lack of cortisol elevation in the d-cDOpH + A treatment compared to d-cDOpH treatment, at either temperature (Fig. 2b and d), demonstrates that algicide exposure does not interact with these potential multiple stressors to impact cortisol level in *M. menidia*.

## Discussion

Harmful dinoflagellates are responsible for massive fish kills worldwide through production of ichthyotoxic compounds, mechanical damage to gills or because of hypoxic or anoxic conditions resulting from the demise of dinoflagellate blooms (reviewed by Anderson et al. 2021). Efforts to control dinoflagellate blooms, including the application of algicidal chemicals (e.g., copper sulfate), or physical control methods (e.g., clay flocculation), can have negative effects on non-target species (reviewed by Sellner and Rensel 2018). Application of algicidal products from naturally occurring bacteria is considered an environmentally friendly approach to control algal blooms (reviewed by Coyne et al. 2022; Kan et al. 2011), since these bacteria are already present in the environment. However, the effects of algicides on non-target species are not always evaluated, and then only rarely in the context of other potential stressors (e.g., Simons et al. 2021).

Here, we examined whether exposure to the bacterial algicide IRI-160AA caused a significant cortisol stress response in *F. heteroclitus* and *M. menidia* and whether other potential co-occurring environmental stressors (diel-cycling hypoxia and/or pH, at two temperatures) exacerbate potential impact of the algicide. Our results demonstrate that exposure to IRI-160AA does not significantly affect cortisol levels in either species, at either temperature tested, regardless of whether algicide exposure occurs independently or with diel-cycling hypoxia and/or pH in a multiple stressor context.

There are known sublethal impacts of diel-cycling hypoxia and pH on growth, survival, respiration, and behavioral responses of *F. heteroclitus* and *M. menidia* and other fish that live in salt marsh estuaries (Stierhoff et al. 2003; Miller et al. 2016; Dixon et al. 2017; Targett et al. 2019; Morrell and Gobler 2020). These stressors have been shown to elicit a cortisol stress response in fish. Petochi et al. (2011) reported higher plasma cortisol during acute exposure to high pCO_2_ (low pH) in juvenile European sea bass (*Dicentrarchus labrax*). Simons and Targett (submitted) provided the first evidence of a cortisol stress response in *F. heteroclitus* and *M. menidia* exposed to the d-cDOpH treatment in the present experiments by focusing on DO and pH effects, without algicide treatments, using a combined significance test across species and temperatures.

Measurement of cortisol stress response to study algicide effects on fish has the advantage of short response-time. In the experiments reported here, the mean time between exposure to algicide (and associated controls) and when fish were removed and anesthetized was 1 h:45 min (± 0.01 SE), well within the time frame for cortisol release in fish. It is noteworthy that the work reported here showed no cortisol elevation in any d-cDOpH + A treatments compared with d-cDOpH treatments, providing further evidence that algicide did not impact cortisol in these fish, even within this multiple stressor context.

Results of this investigation, along with other reports showing no negative effects of algicide IRI-160AA on non-target phytoplankton and heterotrophic protists (Hare et al. 2005; Pokrzywinski et al. 2012; Tilney et al. 2014; Grasso 2019) or oyster and copepod larvae and adults, and only transient effects on crab larvae (Simons et al. 2021), support application of algicide IRI-160AA as an environmentally friendly method to control harmful algal blooms in coastal environments. Further studies should examine responses of other fish species in salt marsh estuaries and fish in other environments to determine the generality of these results to other coastal ecosystems at risk of harmful dinoflagellate blooms. Data variability inherent in assessment of cortisol levels suggests that further research include larger sample sizes than were possible here.

## Supplementary Information

Below is the link to the electronic supplementary material.

Supplementary file1 (DOCX 363 KB)

## Data Availability

Plasma cortisol and system DO and pH data that support the findings of this study have been published in Zenodo (https://zenodo.org/badge/DOI/10.5281/zenodo.13835437.svg).
