# Supplementary material for: Bacteria-Produced Algicide for Field Control of Toxic Dinoflagellates Does Not Cause a Cortisol Stress Response in Two Estuarine Fish Species
Source: Mar Biotechnol (NY). 2025 Jan 14;27(1):29. doi: 10.1007/s10126-024-10383-z (PMC11732778; doi:10.1007/s10126-024-10383-z)

**Bacteria-Produced Algicide for Field Control of Toxic Dinoflagellates Does Not Cause a Cortisol Stress Response in Two Estuarine Fish Species**

Victoria E Simons^1^, Timothy E Targett^*1^, Patrick M. Gaffney^1^, Kathryn J. Coyne ^ǂ1^

**Online Resource 1** Schematic of one of the five recirculating aquarium systems (~400 L) used to create the dissolved oxygen, pCO_2_, and associated pH conditions for experiments reported in this paper. This side view shows one of the two rows of five ~20 L polyethylene tanks (10 total) each system contained. Blue arrows indicate direction of water flow.


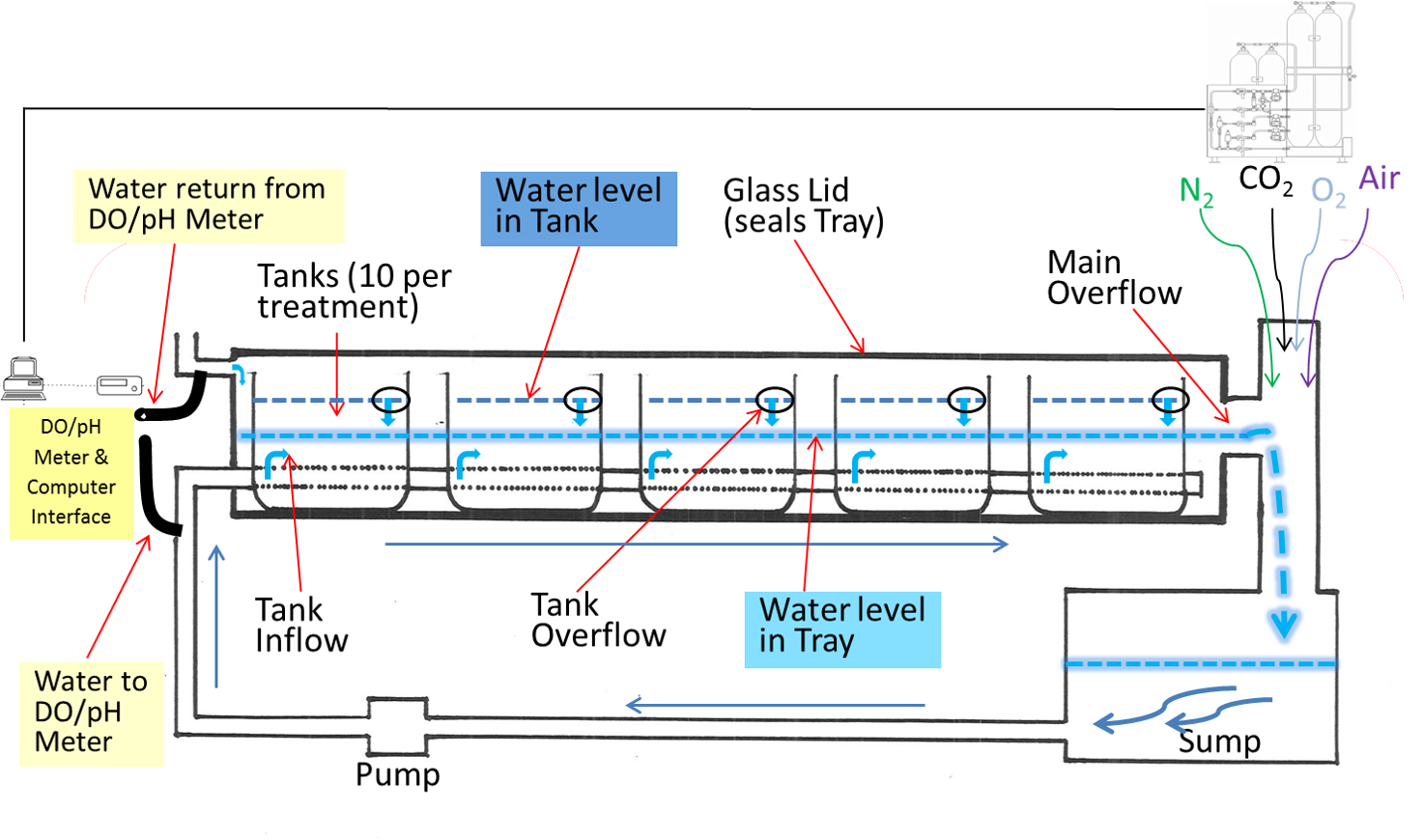

Supplement: Supplementary file 1 — Supplementary file1 (DOCX 363 KB) [file 10126_2024_10383_MOESM1_ESM.docx]
